# Salivary Profile in Oral Submucous Fibrosis: A Scoping Review

**DOI:** 10.1055/s-0044-1788711

**Published:** 2024-08-05

**Authors:** Fatma Yasmin Mahdani, Ajiravudh Subarnbhesaj, Nurina Febriyanti Ayuningtyas, Meircurius Dwi Condro Surboyo, Reiska Kumala Bakti, Desiana Radithia, Dimas Bayu Paramananda, Ina Indriyani, Fatimah Fauzi Basalamah

**Affiliations:** 1 Department of Oral Medicine, Faculty of Dental Medicine, Universitas Airlangga, Surabaya, Indonesia; 2 Division of Oral Diagnosis, Department of Oral Biomedical Science, Faculty of Dentistry, Khon Kaen University, Khon Kaen, Thailand; 3 Bachelor of Dental Science, Faculty of Dental Medicine, Universitas Airlangga, Surabaya, Indonesia; 4 Oral Medicine Specialist Study Program, Faculty of Dental Medicine, Universitas Airlangga, Surabaya, Indonesia

**Keywords:** saliva, oral submucous fibrosis, oral potentially malignant disorder, oral cancer

## Abstract

Diagnosing oral submucous fibrosis (OSMF) is invariably challenging. The disease can be detected after reaching its final stage and requires complex treatment. Changes in its salivary profile can be used as a reference to see this disorder and as a basis for diagnostic prediction. This study is aimed to analyze the salivary profile as a diagnosis marker in patients with OSMF. The study using Preferred Reporting Items for Systematic Reviews and Meta-analyses was conducted using PubMed, Science Direct, and Scopus databases. A thorough literature search between 1991 and 2023 was performed. Twenty-eight full-text articles were reviewed in detail. Twenty-eight articles were included; a total of 929 patients of OSMF and 826 controls were found. The scoping review showed that levels of salivary protein (including lactate hydrogenase, immunoglobulin G, immunoglobulin A, S1007A protein, 8-hydroxydeoxyguanosine, 8-isoprostane, malondialdehyde, matrix metalloproteinase-12, salivary C-reactive protein, fibrinogen producing factor, salivary miRNA-21, and salivary lipids [cholesterol, high-density lipoprotein, triglyceride) were higher in OSMF. Meanwhile, trace elements (vitamin C, vitamin E, iron, zinc, and magnesium) were lower; only copper was higher in OSMF patients. Alteration in salivary components such as protein, lipid, and trace elements detection can be a basis for providing a noninvasive supportive examination and thus be used as a diagnosis marker of OSMF.

## Introduction

Oral submucous fibrosis (OSMF) is a chronic mucosal disorder included in the Oral Potentially Malignant Disorders (OPMDs) group characterized by progressive inflammation and fibrosis of the submucosal tissue. 1 2 3 The etiology of OSMF is currently unknown, most likely to be multifactorial. However, a study conducted in a rural area of Sindh, Pakistan, noted a higher incidence of more than 90% of OSMF found among consumers of areca nut and related products. 4

The primary diagnostic method for establishing OSMF is a biopsy. Biomarkers of biopsy, such as cytological features, promoter methylation, polymorphism, mRNAs, microRNAs, noncoding RNAs, proteins, and trace elements determine the staging and classification of OSMF. These biomarkers were detected by methylated polymerase chain reaction (PCR), real-time PCR, western blotting, and staining procedures. 5 6 However, the biopsy sometimes is an invasive procedure with low patient acceptance. Liquid biopsy shows the noninvasive detection of components in biofluids, such as blood serum and saliva. A liquid biopsy is a revolutionary approach with significant potential for diagnosis with high patient acceptance, although more supporting data are needed to establish accuracy. Liquid biopsy from salivary samples using biochemical and biomolecular techniques is more stable and sensitive; even low concentrations of free ions, circulating cells, proteins, nucleic acids, and enzymes can be detected in saliva. In recent years, OSMF biomarkers have been identified in blood serum, and saliva, and their application feasibility in diagnosing OSMF has increased. 7 8

In this review, we collect evidence of salivary profile in OSMF patients and analyze the salivary component changes.

## Methods

### Standard of Reporting and PICO Principle

The present scoping review followed the Preferred Reporting Items for Systematic Reviews and Meta-analyses (PRISMA) 2020 guidelines. The studies were identified using the PICO principle: Patients = patients with OSMF, Intervention = method of quantitative analysis of saliva, Comparison = healthy individuals, Outcome = component changes in saliva in patients with OSMF.

### Study Selection

All case–control, cross-sectional, and quasi-experimental studies that evaluate the salivary components in patients of OSMF, compared with a control group, which fulfill the following inclusion criteria, were included. The inclusion criteria were as follows: (a) studies about OSMF; (b) case–control, cross-sectional, and quasi-experimental studies; and (c) studies about salivary components in patients of OSMF.

### Data Sources and Search Strategy

A comprehensive scientific literature search was conducted in December 2021 in the following databases: PubMed (U.S. National Library of Medicine, MD), ScienceDirect (Elsevier, Netherlands), and Scopus Document ( https://www.scopus.com/search/form.uri?display=basic#basic ) for studies published from 1991 to 2023. The search strategy was a combination of the following keywords adapted to each database: [(saliva) AND (oral submucous fibrosis) OR (oral submucous fibroses) OR (oral submucosal fibrosis)].

The exclusion criteria were as follows: (a) studies that were not about OSMF and did not provide a healthy individual as control; (b) review articles, systematic review, and meta-analysis; and (c) studies about salivary properties (pH, volume, viscosity).

All studies obtained from databases searched with the above searching criteria were pooled together and duplicates were removed. The remaining studies were then filtered by reading “title” and “abstract.” Studies that did not meet the inclusion criteria were then excluded at this step. The remaining studies were screened at the final step by thoroughly reading the full text and those that did not meet the inclusion criteria were excluded.

## Results

### Characteristics of Study Included

**Fig. 1:**
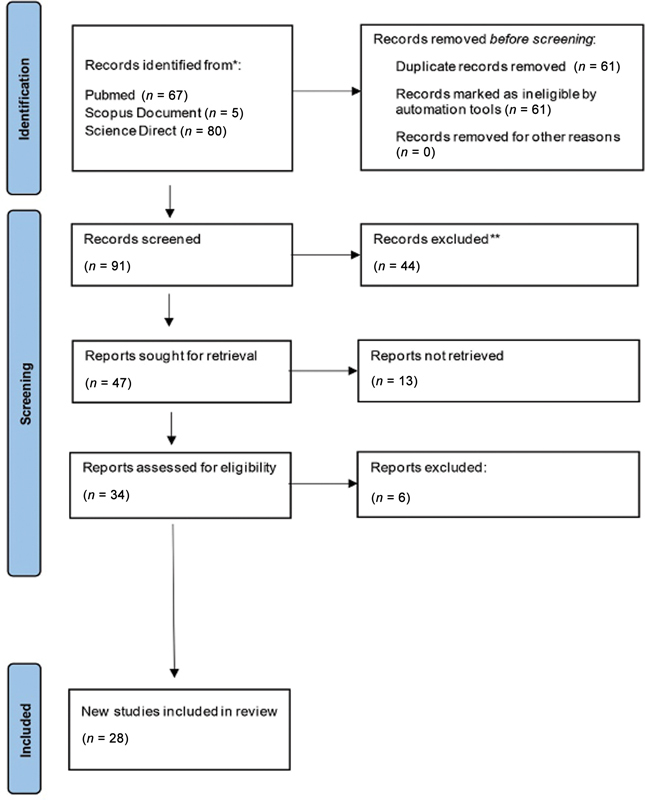
PRISMA flow chart of the literature search study selection. PRISMA, Preferred Reporting Items for Systematic Reviews and Meta-analyses. *Reports the number of records identified from each database (Pubmed, Scopus, and Science Direct). **Many notes excluded by author.

A literature search with the specified keywords in a total of 152 articles was obtained after the initial search using keywords “saliva” and “oral submucous fibrosis”; “saliva” and “oral submucous fibroses”; “saliva” and “oral submucosal fibrosis,” the remaining 91 articles were obtained after removing duplicates. After reviewing the abstracts and titles, 34 articles were selected. Of these, 28 articles were considered for inclusion in the scoping review and 6 were excluded. Data were then collected from 28 articles, and a total of 929 cases of OSMF in patients and 826 controls were found. The PRISMA flowchart of the study search is presented in Fig. 1 and characteristics of studies included in the scoping review are shown in Table 1 .

**Table 1:** Characteristics of studies included in the systematic review

Number	References	Number of patients		Marker	Methods of analysis	Study design	Significance
		Control	OSF				
**1**	Saleem et al 32	30	30	Salivary matrix metalloproteinase-12	ELISA	Cross-sectional study	Higher
**2**	Singh et al 33	25	26	Salivary lipid levels	Cholesterol: enzymatic CHOD-PAP method HDL: direct enzymatic (polyvinyl sulfonic acid/polyethylene-glycol ether) method Triglyceride: enzymatic (GPO-PAP) calorimetric method	Cross-sectional study	Higher
**3**	Raffat et al 26	33	30	S100A7 protein expression	ELISA commercial kit	Cross-sectional study	Higher
**4**	Kallalli et al 20	10	25	Salivary lactate dehydrogenase	ERBA-CHEM 5 semi autoanalyzer	Cross-sectional study	Higher
**5**	Shetty et al 11	50	50	Trace elements in saliva: copper, zinc, and iron	The standardized Cu solutions using the GBC Avanta atom absorption spectrophotometer	Cross-sectional study	Copper: higher Zinc: lower Iron: lower
**6**	Divya and Sathasivasubramanian 23	30	30	Salivary immunoglobulin G and immunoglobulin A	Dade Behring BN ProSpec Nephelometer	Cross-sectional study	Higher
**7**	Ayinampudi and Narsimhan 13	6	5	Salivary copper and zinc levels	Inductively coupled mass spectrometry, Agilent 7500ce	Cross-sectional study	Copper: higher Zinc: lower
**8**	Gupta et al 25	20	20	Immunoglobulin A	Quantia IgA	Cross-sectional study	Higher
**9**	Prasad et al 35	63	61	Expression of salivary miRNA	SYBR Chemistry in an Applied Biosystems Real-Time PCR	Cross-sectional study	Higher
**10**	Ganta et al 31	40	40	Salivary malondialdehyde	Thiobarbituric acid-trichloroacetic acid method	Case–control study	Higher
**11**	Nandakumar et al 28	30	30	Salivary 8-Hydroxydeoxyguanosine	Sandwich ELISA	Case–control study	Higher
**12**	Panda et al 17	40	40	Salivary lactate dehydrogenase	Semiautomatic Analyzer (Accurex-ACCULAB AT300D)	Case–control study	Higher
**13**	Khulbe et al 9	60	60	Salivary copper, zinc, and iron	semiautomated analyzer (ERBA CHEM-5 Plus V2) based on “absorption photometry”	Case–control study	Copper: higher Zinc: lower Iron: lower
**14**	Mantri et al 18	30	30	Salivary lactate dehydrogenase	UV semiautomated spectrophotometer	Case–control study	Higher
**15**	Raffat et al 27	30	30	S100A7 protein expression	Sandwich ELISA	Case–control study	Higher
**16**	Mishra et al 19	20	20	Salivary lactate dehydrogenase	Royato 9200 Chemistry Semi Autoanalyzer	Case–control study	Higher
**17**	Kandasamy et al 22	20	20	Salivary immunoglobulin G and immunoglobulin A	Turbidimetric immunoassay method	Case–control study	Higher
**18**	Sivaramakrishnan et al 21	30	30	Salivary lactate dehydrogenase	LDH assay kit and a UV-visible spectrophotometer (Systronics)	Case–control study	Higher
**19**	Mohammed et al 10	30	30	Copper in saliva	Atomic absorption spectrophotometer method	Case–control study	Higher
**20**	Kode and Karjodkar 12	15	30	Trace elements in saliva: copper, zinc, iron, and magnesium	Atomic absorption spectrometry and a Differential Pulse Anodic Stripping Voltmeter	Case–control study	Copper: higher Zinc: lower Iron: lower Magnesium: lower
**21**	Shetty et al 30	21	65	Salivary MDA	Thiobarbutric acid reactive substances	Case–control study	Higher
**22**	Shetty et al 14	21	65	Micronutrient status in saliva: iron and ascorbic acid (vitamin C) levels	Salivary ascorbic acid: the dintrophenyl hydrazine method salivary iron: the dipyridyl method	Case–control study	Iron: lower Vitamin C: lower
**23**	Patidar et al 24	10	30	Salivary immunoglobulin G and immunoglobulin A	Quantia IgG and IgA turbidometric immunoassay	Case–control study	Higher
**24**	Bhalerao et al 15	22	22	Salivary vitamin C, salivary iron	Ferrozine method and 2-4 dinitrophenylhydrazine method	Case–control study	Vitamin C: lower Iron: lower
**25**	Kaur et al 16	40	40	Salivary 8-hydroxy-2-deoxyguanosine, malondialdehyde, vitamin C, and vitamin E	Receiver operating characteristic analysis	Case–control study	8-OHdG: higher MDA: higher Vitamin C: lower Vitamin E: lower
**26**	Wanjari et al 34	50	50	Fibrin producing factor	The King's method	Case–control study	Higher
**27**	Meera et al 29	10	10	Salivary 8-isoprostane	ELISA procedure	Case–control study	Higher
**28**	Uppal et al 36	30	30	Salivary C-reactive protein	CRP-Turbilatex method, a quantitative turbidimetric method	Quasi-experimental study	Higher

Abbreviations: 8-OHdG, 8-Hydroxydeoxyguanosine; ELISA, enzyme-linked immunosorbent assay; HDL, high-density lipoprotein; IgA, immunoglobulin A; IgG, immunoglobulin G; LDH, lactate dehydrogenase; MDA, malondialdehyde.

### Salivary Profile Analysis

Lactate dehydrogenase (LDH), salivary immunoglobulin G (IgG), salivary immunoglobulin A (IgA), S1007A, and salivary miRNAs 21 were significantly increased in patients with OSMF compared with other healthy individuals. 8-Hydroxydeoxyguanosine (8-OHdG) and 8-isoprostane in saliva showed an average increase from typical to OSMF to Oral Squamous Cell Carcinoma (OSCC) but not statistically significant. Malondialdehyde (MDA) levels were significantly increased in OSMF patients with clinical stage progress. Matrix metalloproteinase-12 (MMP-12) was markedly increased in patients with OSMF compared with other healthy individuals, salivary C-reactive protein (CRP) levels were increased in malignant conditions or OSMF patients, fibrinogen-producing factor (FPF) could indicate an increase in saliva levels in OSMF patients. Lipids such as cholesterol, high-density lipoprotein (HDL), and triglyceride (TG) showed a rise in salivary lipid levels in OSMF patients compared with healthy individuals. Vitamin and trace elements, such as vitamin C, vitamin E, iron, zinc, and magnesium were lowered in patients with OSMF compared with the control, presented in Tables 2 3 4 5 6 7 8 9 10 .

**Table 2:** The salivary *mineral* profile in included study

Number	References	Number of patients		Marker	Methods of analysis	Staging of OSF	Method of collecting saliva	Significance
		Control	OSF					
1	Khulbe et al 9	60	60	Copper	Absorption photometry	There was 1 patient of stage I, 25 patients of stage II, 23 patients of stage III, and 11 patients of stage IV	Unstimulated whole saliva	*p* < 0.05
				Zinc	Absorption photometry	There was 1 patient of stage I, 25 patients of stage II, 23 patients of stage III, and 11 patients of stage IV	Unstimulated whole saliva	*p* < 0.05
				Iron	Absorption photometry	There was 1 patient of stage I, 25 patients of stage II, 23 patients of stage III, and 11 patients of stage IV	Unstimulated whole saliva	*p* < 0.05
2	Mohammed et al 10	30	30	Copper	Atomic absorption spectrophotometer method	There were 15 patients of stage I, 6 patients of stage II, and 9 patients of stage III	Unstimulated whole saliva	*p* < 0.005
3	Shetty et al 11	50	50	Copper	The standardized Cu solutions using the GBC Avanta atom absorption spectrophotometer	Not mentioned	Unstimulated whole saliva	*p* = 0.001
				Zinc	The standardized Cu solutions using the GBC Avanta atom absorption spectrophotometer	Not mentioned	Unstimulated whole saliva	*p* = 0.001
				Iron	The standardized Cu solutions using the GBC Avanta atom absorption spectrophotometer	Not mentioned	Unstimulated whole saliva	*p* = 0.001
4	Kode and Karjodkar 12	15	30	Copper	Atomic absorption spectrophotometer method	Stage III	Unstimulated whole saliva	*p* = 0.01
				Zinc	Atomic absorption spectrophotometer method	Not mentioned	Unstimulated whole saliva	*p* = 0.01
				Iron	Atomic absorption spectrophotometer method	Not mentioned	Unstimulated whole saliva	*p* = 0.01
				Magnesium	Atomic absorption spectrophotometer method	Not mentioned	Unstimulated whole saliva	*p* = 0.01
5	Ayinampudi and Narsimhan 13	6	5	Copper	ICP-MS	Not mentioned	Unstimulated whole saliva	*p* < 0.01
				Zinc	ICP-MS	Not mentioned	Unstimulated whole saliva	*p* < 0.05
6	Shetty et al 14	21	65	Iron	Dipyridyl method	There were 22 patients of stage I, 20 patients of stage II and 23 patients of stage III.	Unstimulated whole saliva	*p* < 0.001
7	Bhalerao et al 15	22	22	Iron	Ferrozine method	there were 3 patients of stage I, 13 were of stage II and 6 were of stage III.	Unstimulated whole saliva	*p* < 0.001

Abbreviation: ICP-MS, inductively coupled mass spectrometry.

**Table 3:** The salivary *lactate hydrogenas* *e* profile in included study

Number	References	Number of patients		Marker	Methods of analysis	Staging of OSF	Method of collecting saliva	Significance
		Control	OSF					
1	Panda et al 17	40	40	LDH	Semiautomatic Analyzer (Accurex-ACCULAB AT300D)	Not mentioned	Unstimulated whole saliva	*p* < 0.05
2	Mantri et al 18	30	30	LDH	UV semiautomated spectrophotometer	Not mentioned	Unstimulated whole saliva	*p* < 0.001
3	Mishra et al 19	20	20	LDH	Royato 9200 Chemistry Semi Autoanalyzer	Not mentioned	Unstimulated whole saliva	*p* < 0.05
4	Kallalli et al 20	10	25	LDH	ERBA-CHEM 5 semi autoanalyzer	Not mentioned	Unstimulated whole saliva	*p* < 0.0009
5	Sivaramakrishnan et al 21	30	30	LDH	LDH assay kit and a UV-visible spectrophotometer	Stage II and stage III	Unstimulated whole saliva	*p* < 0.001

Abbreviation: LDH, lactate hydrogenase.

**Table 4:** The salivary *immunoglobulin* profile in included study

Number	References	Number of patients		Marker	Methods of analysis	Staging of OSF	Method of collecting saliva	Significance
		Control	OSF					
1	Kandasamy et al 22	20	20	IgG	Turbidimetric immunoassay method	There were 3 patients of stage I, 10 patients of stage II, and 7 patients of stage III	Unstimulated whole saliva	*p* < 0.001
				IgA	Turbidimetric immunoassay method	There were 3 patients of stage I, 10 patients of stage II, and 7 patients of stage III	Unstimulated whole saliva	*p* < 0.001
2	Divya and Sathasivasubramanian 23	30	30	IgG	Dade Behring BN ProSpec Nephelometer	Not mentioned	Unstimulated whole saliva	*p* = 0.38
				IgA	Dade Behring BN ProSpec Nephelometer	Not mentioned	Unstimulated whole saliva	*p* = 0.85
3	Patidar et al 24	10	30	IgG	Quantia turbidometric immunoassay	Stage II: highly significant, stage III: significant	Unstimulated whole saliva	*p* < 0.01
				IgA	Quantia turbidometric immunoassay	Stage IV: significant	Unstimulated whole saliva	*p* < 0.01
4	Gupta et al 25	20	20	IgA	Quantia IgA	There were 10 patients of stage I, 6 patients of stage II, and 4 patients of stage III	Unstimulated whole saliva	Not mentioned

Abbreviations: IgA, immunoglobulin A; IgG, immunoglobulin G.

**Table 5:** The salivary *vitamin* profile in included study

Number	References	Number of patients		Marker	Methods of analysis	Staging of OSF	Method of collecting saliva	Significance
		control	OSF					
1	Shetty et al 14	21	65	Vitamin C	Dintrophenyl hydrazine method	There were 22 patients of stage I, 20 patients of stage II, and 23 patients of stage III	Unstimulated whole saliva	*p* < 0.001
2	Bhalerao et al 15	22	22	Salivary vitamin C	2,4- Dinitrophenylhydrazine	There were 3 patients of stage I, 13 were of stage II, and 6 were of stage III	Unstimulated whole saliva	*p* < 0.01
3	Kaur et al 16	40	40	Vitamin A	Estimated by HPLC	Not mentioned	Unstimulated whole saliva	*p* < 0.005
				Vitamin C	Estimated by HPLC	Not mentioned	Unstimulated whole saliva	*p* < 0.005

**Table 6:** The salivary *malondialdehyde* profile in included study

Number	References	Number of patients		Marker	Methods of analysis	Staging of OSF	Method of collecting saliva	Significance
		Control	OSF					
1	Ganta et al 31	40	40	MDA	Thiobarbituric acid-trichloroacetic acid method	There were 10 patients of stage I, 16 were of stage II and 14 were of stage III	Unstimulated whole saliva	*p* < 0.05
2	Shetty et al 30	21	65	MDA	Thiobarbutric acid reactive substances	Stage III	Unstimulated whole saliva	*p* < 0.001
3	Kaur et al 16	40	40	MDA	Thiobarbutric acid reaction	Not mentioned	Unstimulated whole saliva	*p* < 0.005

Abbreviation: MDA, malondialdehyde **.**

**Table 7:** The salivary *lipid* profile in included study

Number	References	Number of patients		Marker	Methods of analysis	Staging of OSF	Method of collecting saliva	Significance
		control	OSF					
1	Singh et al 33	25	26	Cholesterol	Enzymatic CHOD-PAP method	Not mentioned	Unstimulated whole saliva	Not mentioned
				HDL	Direct enzymatic (polyvinyl sulfonic acid/polyethylene-glycol ether) method	Not mentioned	Unstimulated whole saliva	Not mentioned
				Triglyceride	Enzymatic (GPO-PAP) calorimetric method	Not mentioned	Unstimulated whole saliva	Not mentioned

Abbreviation: HDL, high-density lipoprotein.

**Table 8:** The salivary *S100A7* profile in included study

Number	References	Number of patients		Marker	Methods of analysis	Staging of OSF	Method of collecting saliva	Significance
		Control	OSF					
1	Raffat et al 26	33	30	S100A7	ELISA	Stage I	Unstimulated whole saliva	*p* < 0.001
2	Raffat et al 27	30	30	S100A7	Sandwich ELISA	Stage I	Unstimulated whole saliva	*p* < 0.001

Abbreviation: ELISA, enzyme-linked immunosorbent assay.

**Table 9:** The salivary *8-hydroxydeoxyguanosine* profile in included study

Number	References	Number of patients		Marker	Methods of analysis	Staging of OSF	Method of collecting saliva	Significance
		Control	OSF					
1	Kaur et al 16	40	40	8-hydroxy-2-deoxyguanosine	ROC	Not mentioned	Unstimulated whole saliva	*p* < 0.005
2	Nandakumar et al 28	30	30	8-OHdG	Sandwich ELISA	Not mentioned	Unstimulated whole saliva	*p* < 0.0001

Abbreviations: 8-OHdG, 8-hydroxydeoxyguanosine; ELISA, Abbreviation: ELISA, enzyme-linked immunosorbent assay; ROC, receiver operating characteristic.

**Table 10:** The other salivary profile in included study

Number	References	Number of patients		Marker	Methods of analysis	Staging of OSF	Method of collecting saliva	Significance
		Control	OSF					
1	Saleem et al 32	30	30	MMP-12	ELISA	Stage IV	Unstimulated whole saliva	*p* < 0.001
2	Meera et al 29	10	10	Salivary 8-isoprostane	ELISA	Not mentioned	Unstimulated whole saliva	*p* < 0.853
3	Prasad et al 35	63	61	Salivary miRNA 21	Real-time PCR	There were 3 patients of stage I, 28 patients of stage II, 24 patients of stage III, and 6 patients of stage IV	Unstimulated whole saliva	*p* < 0.001
4	Uppal et al 36	30	30	CRP	Quantitative turbidimetric method	Not mentioned	Unstimulated whole saliva	*p* < 0.001

Abbreviations: CRP, C-reactive protein; ELISA, enzyme-linked immunosorbent assay; MMP-12, matrix metalloproteinase-12.

## Discussion

OSMF is a chronic mucosal disease characterized by progressive inflammation and fibrosis of submucosal tissue. OSMF can be classified as an OPMD, which can be transformed into a malignant disease so that it prompts early detection to minimize its transformation being malignant. Diagnosis staging of OSMF is based on clinical signs and symptoms that include burning sensation, pain, and ulceration and based on restriction in mouth opening, and grading of OSMF is based on histopathology grading. 1 2 3

**Fig. 2:**
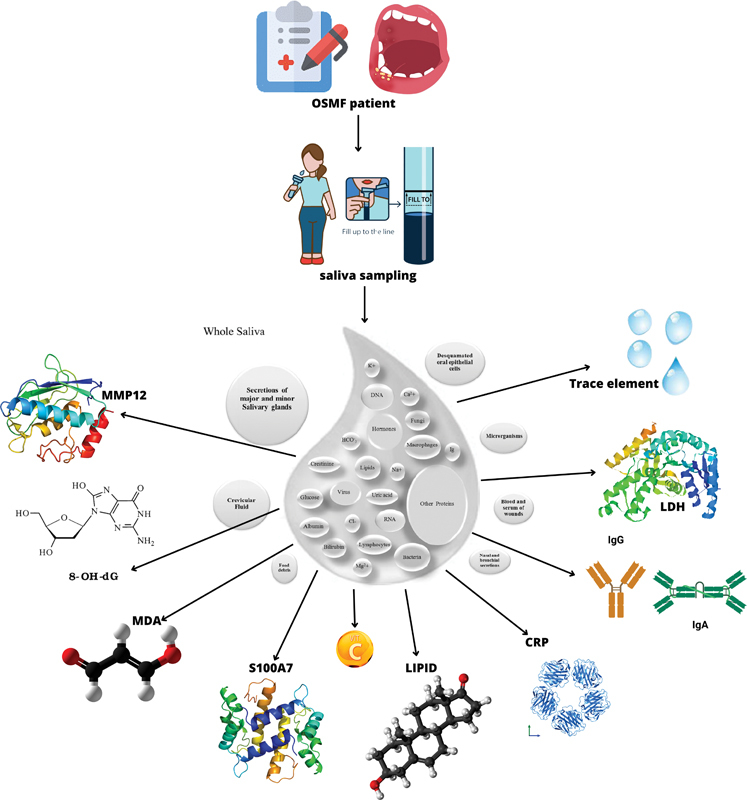
Salivary profile as diagnostic marker in patient with OSMF. 8-OHdG, 8-hydroxydeoxyguanosine; CRP, C-reactive protein; IgA, immunoglobulin A; IgG, immunoglobulin G; LDH, lactate hydrogenase; MDA, malondialdehyde; MMP-12, matrix metalloproteinase-12; OSMF, oral submucous fibrosis.

Unstimulated whole saliva can be chosen because it is a complex mix of salivary content referring to the complex mix of saliva, gingival crevicular fluid, oral bacteria and food debris, and pieces of chemicals or medicaments. Salivary component analysis can be used as an OSMF marker for predicting diagnosis. The whole unstimulated and stimulated saliva were used as OSMF markers considering they are noninvasive supportive examinations. This investigation discloses that most included studies reported on LDH, vitamins, trace elements, and lipids, furthermore revealed the presence of MMP-12, IgA, IgG, CRP, MDA, S1007A, 8-OHdG, and miRNA-21 as presented in Fig. 2 .

OSMF must be detected in advance as early prevention of malignancy. OSMF biomarkers in saliva can also be indicated by vitamin C, vitamin E, and mineral content such as copper, zinc, iron, and magnesium. Regarding studies, those focused on minerals in saliva, predominantly represented by seven researches, studied the vitamins such as vitamin C, vitamin A, and vitamin E. Three research journals proposed that ascorbic acids or vitamin C and vitamin E can be biomarkers for OSMF since they can potentially protect cytosolic components and cell membranes from oxidative damage. Salivary ascorbic acid levels consistently depressed with the development of histopathological assessment of OSMF. In addition to low levels of vitamin C and vitamin E, the average levels of salivary zinc, magnesium, and iron in OSMF patients were also lower compared with the healthy individual group. Conversely, the copper mineral was increased in OSMF patients though a study stated that the level of copper has depressed. Minerals can be oral biomarkers for OSMF because the trace elements are anticancer agents capable of regulating various biological mechanisms. Many researchers have observed the relationship between trace elements and cancer mortality. 9 10 11 12 13 14 15 16

LDH level in saliva can also be a candidate for OSMF biomarkers as it involves the oral epithelium's structure. Therefore, several pathological occurrences in the oral epithelium can cause alteration in salivary LDH concentrations. LDH is present in all normal cells and is considered a metabolic enzyme released extracellularly upon cell death. Precancerous and oral cancer patients have higher LDH levels compared with normal patients associated with cell necrosis and tissue damage. Five research studies suggested elevated salivary LDH levels in patients with OSMF than in healthy individuals. 17 18 19 20 21

Some studies proposed OSMF as an autoimmune disorder because of its incidence with no history of irritant usage and hereditary disease, but a noticeable immunological change. Salivary antibodies such as IgG and IgA are commonly screened humoral immune components. Four studies suggested salivary IgG and IgA levels were statistically found to be markedly raised in OSMF patients. On the contrary, Total Salivary Protein (TSP) was reduced in OSMF patients compared with the control group. Consequently, the value uncertainty results in limitations in statistical analysis. 22 23 24 25

Salivary protein S100A7 binds directly to the receptor for advanced glycation end products and promotes inflammation. As well, S100A7 overexpression has been reported in several cancers. 26 In the conducted research, it can be seen that the OSMF stage I group was compared with healthy individuals. Two studies suggests patients with OSMF have higher salivary S100A7 levels compared with healthy individuals, and it is possibly applied as a surrogate measure to identify high-risk subjects for OSMF. 26 27

Salivary 8-OHdG can be observed through a relatively noninvasive, simple, and efficient methodology to monitor oxidative stress in subjects with OPMD which can be used to identify OSMF. There was a clear correlation between an increase in the number of pocket years in OSMF patients and an increase in 8-OhDG levels, both by sandwich ELISA and receiver operating characteristic methods. 16 28 In addition to the 8-OHdG level, it can be seen that the level of 8-isoprostane in saliva showed an average increase from typical to OSMF to OSCC but was not statistically significant. 29

Salivary MDA is a toxic compound that reacts with DNA to form covalent bonds with deoxyadenosine and deoxyguanosine, an event resulting in a mutagenic transformation in DNA by altering its chemical behavior and possibly contributing to carcinogenesis and mutagenesis. Three published researches conducting the thiobarbituric acid-trichloroacetic acid method proposed that salivary MDA levels peaked in OSMF patients with clinical stage progress. 16 30 31

Salivary MMP-12 is a valuable prognostic biomarker in rare and aggressive tumors due to its functional properties and role in tissue-destructive diseases. MMP-12 is likely used as a biomarker for various oral diseases. Furthermore, it can potentially detect the presence of premalignant developments, including tumor growth, migration, invasion, and tumor metastasis. A statistically significant rise in MMP-12 expression was observed in OSMF and OSCC groups compared with healthy individuals. 32

Lipid levels in saliva can be an alternative to serum lipid levels in identifying OSMF. A study suggested a strong relationship between salivary and serum lipid levels. Serum lipid levels play an essential role in detecting the initiation of precancer and oral cancer, explicitly modifying cell wall integrity, thus leading to cell wall transformation or carcinogenesis. Recent studies have shown that salivary lipid levels are plausible to be used as an indicator of serum lipid levels and a noninvasive technique for measuring serum lipid levels. A report found an increase in salivary lipid levels such as cholesterol, HDL, and TG in OSMF patients compared with healthy individuals. 33

FPF is produced by thrombin-like fibrinogen in saliva, entering the submucosal zone of the oral cavity, and acting on fibrinogen, which later creates local fibrosis. The presence of FPF in saliva may be directly mitogenic to fibroblasts or may lead to fibrin formation by acting on fibrinogen. The results revealed an accumulation of FPF in the saliva of OSMF patients so that it can be used as a biomarker as for an early sign of OSMF. 34

Salivary miRNA-21 can be used as a potential biomarker in detecting oral precancers since miRNA-21 is a tumor suppressor gene in several cell signaling pathways crucial for carcinogenesis. Its excellent stability and resistance to degradation make it the best candidate as a cancer biomarker. It was reported an upregulation of salivary miRNA-21 in OSMF patients compared with healthy individuals. 35

Salivary CRP belongs to an acute phase protein biomarker because of its increasing level in inflammatory conditions. Along with inflammation, CRP can also be found in malignancies. OPMD is a malignant condition because CRP can be found in saliva even though in a very small amount. Hence, CRP in OPMD is relevant considering its levels increase in malignant conditions. 36

The main limitation of this study is the absence of studies providing histopathology examination data to picture the stage and progress of severity of OSMF as well as information on age, gender, ethnicity, or socioeconomic status of the participants. Furthermore, we need more studies with larger samples involving different ages, genders, ethnicities, or socioeconomic statuses of the participants analyzing the salivary component that later can be used as a proper marker of OSMF for predicting its diagnosis. It is still a topic of research and in the clinical world, so it is still being developed. Salivary components in OSMF patients showed alteration in components which might serve as a diagnosis prediction, however further studies about histopathology examination to determine the stage of OSMF are still needed to predict diagnosis of OSMF.

## Conclusion

This review suggests a considerable alteration of salivary profile in OSMF, marked by elevated inflammatory markers and mediators, such as LDH, IgG, IgA, S1007A protein, 8-OHdG, 8-isoprostane, MDA, MMP-12, copper, salivary lipids (cholesterol, HDL, TG), FPF, salivary miRNA-21, and CRP in patients with OSMF compared with control. Reversing them, levels of vitamin and trace elements were depressed. The salivary profile can be developed by providing a noninvasive supportive examination and diagnostic marker for patients with OSMF. This condition encourages further research using saliva as a diagnostic marker in OSMF.
